# Development and Optimisation of an HPLC–MS/MS Workflow for Profiling Selenium and Sulphur Amino Acids in Soybean Leaves and Investigation of Se–S Metabolic Interactions

**DOI:** 10.3390/molecules31111780

**Published:** 2026-05-22

**Authors:** Xiaohui Cai, Jun Men, Qingwu Yang, Yili Hu, Zhixian Qiao

**Affiliations:** 1 School of Modern Industry for Selenium Science and Engineering, Wuhan Polytechnic University, Wuhan 430048, China; 19853506560@163.com (X.C.); 18074604493@163.com (Q.Y.); 2 Analysis and Testing Center, Institute of Hydrobiology, Chinese Academy of Sciences, Wuhan 430072, China; menjun@ihb.ac.cn

**Keywords:** seleno-amino acids, HPLC-MS/MS, multiple reaction monitoring, ultrasound-assisted extraction, soybean, selenium biofortification, selenium–sulfur interaction

## Abstract

A derivatisation-free HPLC–MS/MS method was developed and validated for the simultaneous quantification of selenium- and sulphur-containing amino acids in soybean leaves, and applied to a 3 × 3 factorial hydroponic experiment probing selenium–sulphur metabolic interactions. The method resolves five biologically informative analytes (Cys_2_, SeCys_2_, MeSeCys, Met, SeMet) within 1.5 min through multiple reaction monitoring (MRM). Ultrasound-assisted extraction (UAE) of the free fraction was jointly optimised for both analyte classes by the response-surface methodology; enzymatic hydrolysis of the extraction residue recovered the protein-bound fraction on the same platform. Limits of detection ranged from 0.036 to 0.556 µg L^−1^, intra-day relative standard deviations were below 5%, and spike recoveries fell between 92.3 and 117.4%. Free SeAA and SAA pools were negatively correlated across the nine treatments (R^2^ = 0.83), consistent with competitive Se–S assimilation, whereas bound pools were positively correlated (R^2^ = 0.89), reflecting proportional protein-level incorporation. A regime of 1–5 mM of sulphate with 20 µM of selenite yielded the highest bound organo-Se with near-normal growth, providing leaf-level evidence that may inform future seed-focused studies aimed at Se-enriched soy-protein ingredient development.

## 1. Introduction

Selenium (Se) is an essential micronutrient whose biological activity depends critically on its chemical form. Organoselenium species, notably selenomethionine (SeMet) and Se-methylselenocysteine (MeSeCys), are substantially more bioavailable than the inorganic oxyanions selenite and selenate and serve as principal substrates for selenoprotein biosynthesis, mediating antioxidant defence, thyroid hormone activation, and immune regulation [1,2,3]. Dietary Se deficiency remains a widespread public-health concern across Se-poor agricultural regions [4], and climate-driven losses of soil organic carbon are projected to push approximately one billion people below adequate intake thresholds [5]. Biofortification of food crops with organoselenium has therefore emerged as a scalable intervention [6,7] for which reliable quantification of individual Se species is indispensable to evaluate biofortified products and to elucidate the metabolic fate of Se in planta [8,9].

The metabolic behaviour of Se in plants is mechanistically inseparable from that of sulphur (S), its Group VIA congener. Se oxyanions enter root cells through sulphate transporters and are converted to selenocysteine and SeMet through the same enzymatic cascade that processes cysteine (Cys) and methionine (Met) [10,11,12]. Sulphate supply therefore modulates Se speciation at the whole-plant level, as demonstrated in Arabidopsis [13], wheat [14,15], and a range of Se-accumulating species [16]. Rigorous Se-S investigations accordingly require both analyte classes to be quantified simultaneously under identical preparative and detection conditions [17]. A further layer of complexity is the partitioning of seleno-amino acids (SeAAs) and sulphur amino acids (SAAs) between a soluble-free pool, reflecting short-term metabolic flux, and a protein-bound pool, recording longer-term accumulation. The two fractions often respond differently to Se and S supply, carry distinct mechanistic implications, and are obscured when only total amino acid content is reported.

Several platforms address SeAA and SAA analysis in plant materials. HPLC coupled to inductively coupled plasma mass spectrometry (HPLC–ICP-MS) provides high sensitivity and element-specific detection and remains the reference platform for plant Se speciation [18,19], yet it monitors elemental Se rather than intact molecular ions and cannot resolve Se species from their S analogues in a single run. Classical cation-exchange chromatography with post-column ninhydrin derivatisation quantifies SAAs reliably but lacks selectivity for trace SeAAs in complex plant matrices [20]. Fluorescence-labelling reversed-phase HPLC has been adapted for joint SeAA–SAA detection [21], although derivatisation steps introduce procedural variability and incomplete labelling of labile Se species. HPLC coupled to electrospray tandem mass spectrometry (HPLC-ESI-MS/MS) in multiple reaction monitoring (MRM) mode offers a derivatisation-free alternative with high analyte selectivity on instrumentation more widely available than ICP-MS [19]. Existing MRM-based assays for plant Se, however, have only targeted SeMet alone [22] or SeMet together with Met [23] and have not been validated for leafy tissue, where co-extracted chlorophylls, polyphenols, and sugars challenge both ionisation and extraction recovery. A derivatisation-free MRM workflow covering a broader panel of SeAAs and SAAs in a single chromatographic run, with demonstrated matrix tolerance in leaf tissue, thus remains an unmet analytical need.

Soybean (*Glycine max*) is well suited to the development of such a workflow. It is a principal global source of dietary plant protein (~40% seed protein), and soy protein is listed as an approved carrier for Se fortification under the Chinese National Food Safety Standard GB 14880-2012 [24]. Recent work has begun to characterise Se speciation in biofortified soybean grains [25], but parallel quantification of free and protein-bound SeAAs and SAAs in soybean leaves—the principal site of Se assimilation and S coupling—has not been reported. The 11S globulin (glycinin) fraction preferentially accumulates SeMet regardless of Se source, with SeMet representing >80% of the Se in this fraction [26]. Adjusting the Se-to-S supply ratio provides an agronomic means of regulating Se speciation in this crop, because selenite and sulphate converge on shared assimilation enzymes downstream of uptake [10,11,12,14]. This study develops and validates a derivatisation-free HPLC–MS/MS workflow for the simultaneous quantification of five SeAAs and SAAs [cystine (Cys_2_), selenocystine (SeCys_2_), MeSeCys, Met and SeMet] in free and protein-bound fractions of soybean leaves, and applies it to a 3 × 3 factorial hydroponic experiment combining three selenite with three sulphate supply levels. We hypothesised that free SeAA and SAA pools would respond reciprocally to selenite supply, reflecting competitive flux through shared assimilation enzymes, whereas protein-bound pools would co-accumulate in approximate proportion, consistent with non-selective translational incorporation of SeMet at Met residues. Under this model, an intermediate selenite concentration should maximise bound organo-Se without phytotoxic suppression of protein synthesis. Response-surface optimisation of ultrasound-assisted extraction (UAE) for the free fraction, enzymatic hydrolysis of the UAE residue for the bound fraction, and a unified chromatographic platform together produce an analytically balanced dataset in which free and bound pools can be compared within each biological replicate, providing quantitative evidence of Se-S metabolic interaction and identifying leaf-level cultivation conditions whose relevance to Se-enriched soy-protein ingredient development requires confirmation at the seed level.

## 2. Results

### 2.1. HPLC–MS/MS Method Development

**Figure 1:**
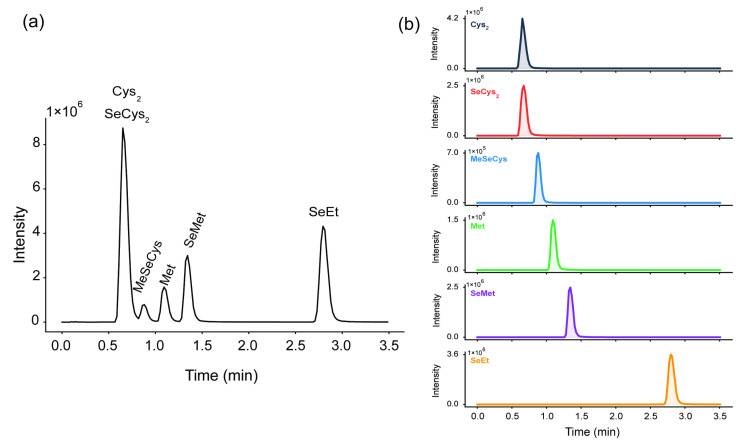
Representative HPLC–MS/MS chromatograms. (**a**) Total ion current chromatogram. (**b**) Individual extracted ion chromatograms of a mixed standard at 100 µg L^−1^: Cys_2_ (*m*/*z* 241.0 → 120.0, t_R_ = 0.65 min), SeCys_2_ (*m*/*z* 335.0 → 246.0, t_R_ = 0.67 min), MeSeCys (*m*/*z* 184.0 → 139.0, t_R_ = 0.88 min), Met (*m*/*z* 150.0 → 104.0, t_R_ = 1.09 min), SeMet (*m*/*z* 198.0 → 109.0, t_R_ = 1.34 min), and SeEt (*m*/*z* 212.0 → 123.0, t_R_ = 2.79 min).

Six selenium- and sulphur-containing amino acids [Cys_2_, SeCys_2_, MeSeCys, Met, SeMet, and selenoethionine (SeEt)] were resolved on the BEH C18 column under the optimised gradient (Figure 1). The five quantitative targets (Cys_2_, SeCys_2_, MeSeCys, Met, and SeMet) eluted within 1.5 min, with peak-to-peak retention-time differences ≥0.2 min between all pairs except Cys_2_ and SeCys_2_ (0.65 and 0.67 min). Co-elution of this pair did not compromise detection because their precursor ions differed by 94 Da (*m*/*z* 241.0 versus 335.0) and were monitored on non-overlapping MRM channels (Figure 1b, Table S1). Analyte-specific resolution at the mass-spectrometric rather than chromatographic stage is a principal advantage of MRM detection for structurally similar SeAA-SAA pairs.

SeEt eluted at 2.79 min but occurs in soybean leaves below the quantification threshold. The effective quantitative readout is therefore delivered within the first 1.5 min, a practically useful attribute for routine quantification of organic Se in plant tissue. Two MRM transitions were adopted per analyte (Table S1), with declustering potential and collision energy tuned individually by direct infusion. The product ion MS/MS spectra of authentic standards acquired under optimised conditions are provided in Figure S1 and confirm the structural assignment of each transition. The total cycle time including column re-equilibration was 7 min per injection.

### 2.2. Analytical Validation in Soybean Leaf Matrix

Validation data are summarised in Table 1. Calibration curves were linear across the evaluated ranges with R^2^ between 0.9995 and 0.9999. The wider linear range for Met (50–5000 µg L^−1^) accommodates its abundance in leaf tissue, which can exceed SeAA concentrations by more than two orders of magnitude. Limits of detection ranged from 0.036 to 0.556 µg L^−1^ and limits of quantification from 0.121 to 1.852 µg L^−1^. Intra-day relative standard deviations were below 5% at all concentration levels and inter-day values below 8%. Spike recoveries in the pooled leaf matrix fell between 92.3 and 117.4%, within the ±20% window accepted for trace analysis in complex matrices [27]. Matrix effects, quantified from slope ratios of matrix-matched to solvent-only calibrations, ranged from 82.3 to 92.4%, indicating modest ion suppression consistent with co-eluting chlorophyll-derived and polyphenolic species [28]. Matrix-matched calibration was adopted for all subsequent measurements. Stability of the target species through the complete UAE and enzymatic hydrolysis workflows was further verified by individual analyte spike recovery in *Glycine max* and the selenium hyperaccumulator *Cardamine violifolia* (recoveries of 87.4–98.3% for UAE and 86.0–97.6% for enzymatic hydrolysis; Tables S2 and S3), with no detectable signal enhancement on non-spiked qualifier channels. Collectively, inter-day precision and spike recovery represent the dominant sources of measurement uncertainty in this workflow (Table 1), and adoption of matrix-matched calibration compensates for systematic ion suppression, so that residual matrix-effect variability is captured within the precision term rather than constituting an independent uncertainty contributor.

### 2.3. Box–Behnken Optimisation of Ultrasound-Assisted Extraction

**Figure 2:**
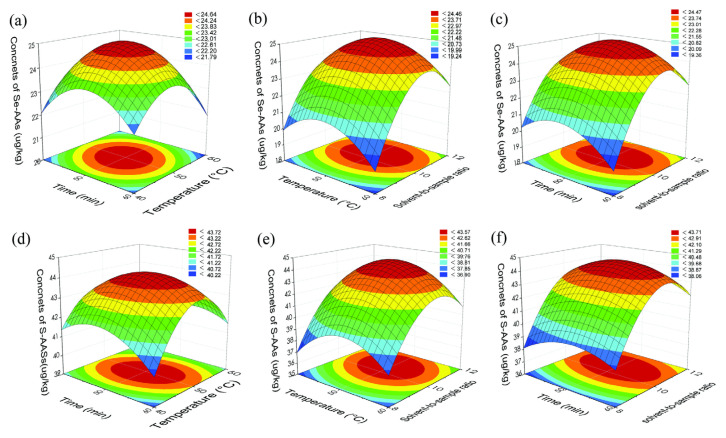
Response surfaces from the Box–Behnken optimisation of UAE. Three-dimensional response surfaces (**a**–**f**) show the effects of ultrasonication time (A, min), temperature (B, °C), and solvent-to-sample ratio (C, mL g^−1^) on the combined desirability-weighted response. The third factor is held at its centre-point value in each panel. The color gradient from blue (low) to red (high) represents the magnitude of the predicted response. Blue indicates lower response values, while red indicates higher response values.

The Box–Behnken design generated 17 experimental runs in which the free organo-Se pool (Y_1_ = SeMet + MeSeCys + SeCys_2_) and the free SAA pool (Y_2_ = Met + Cys_2_) were measured simultaneously (Table S4). Second-order polynomial models fitted to both responses were highly significant (*p* < 0.0001) with non-significant lack-of-fit terms; R^2^ values of 0.9764 and 0.9865 confirmed adequate model fit (Table S5). The solvent-to-sample ratio was the dominant linear factor for both responses; all three factors exhibited significant quadratic terms producing a clear optimum, and no two-factor interactions were significant. Response surfaces for both analyte classes are shown in Figure 2 and Figure S2.

The predicted optima for the two responses converged on nearly identical coordinates. Desirability-function analysis returned a single jointly optimal condition: 10 mL g^−1^ solvent-to-sample ratio, 50 min sonication time, and 50 °C sonication temperature. Triplicate verification yielded Y_1_ = 44.5 ± 0.6 µg kg^−1^ and Y_2_ = 25.1 ± 0.4 µg kg^−1^, within 2% of model predictions.

### 2.4. Morphological Responses to the Se–S Factorial Design

**Figure 3:**
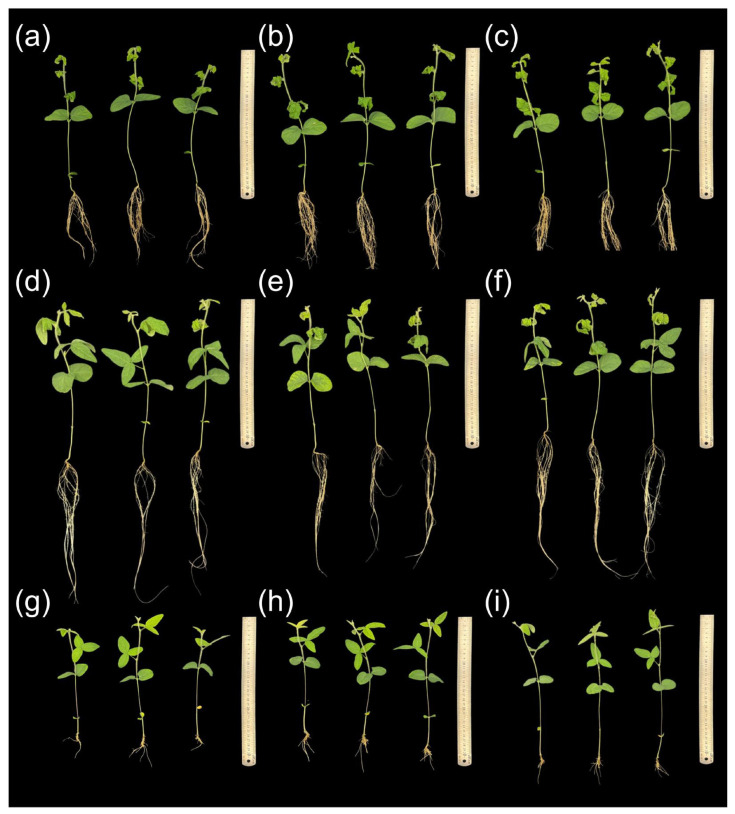
Morphological responses of soybean to the 3 × 3 factorial combination of selenite (0, 20, and 80 µM) and sulphate (0.2, 1.0, and 5.0 mM). Treatment groups are labeled as follows: (**a**) 0 µM Se, 0.2 mM SO_4_^2−^; (**b**) 0 µM Se, 1 mM SO_4_^2−^; (**c**) 0 µM Se, 5 mM SO_4_^2−^; (**d**) 20 µM Se, 0.2 mM SO_4_^2−^; (**e**) 20 µM Se, 1 mM SO_4_^2−^; (**f**) 20 µM Se, 5 mM SO_4_^2−^; (**g**) 80 µM Se, 0.2 mM SO_4_^2−^; (**h**) 80 µM Se, 1 mM SO_4_^2−^; (**i**) 80 µM Se, 5 mM SO_4_^2−^.

Representative photographs from the nine treatment groups are shown in Figure 3, and leaf fresh weight per plant is summarised in Figure S3. At adequate sulphate levels (1 and 5 mM SO_4_^2−^) without Se, plants produced 1.18–1.28 g leaf fresh weight, confirming healthy baseline growth.

Plants at 20 µM selenite across all sulphate levels developed the most extensive root systems, with denser fine-root branching than Se-free controls or high-Se treatments. Leaf biomass was comparable to or modestly greater than controls; the 4–10% increase at 0.2 and 1 mM S was statistically significant (Figure S3), consistent with a nutritional benefit of Se at 20 µM. By contrast, 80 µM selenite reduced leaf fresh weight to 0.45–0.55 g per plant across all sulphate levels (55–65% decrease, *p* < 0.001). These plants displayed shortened stems, sparse root branching, and chlorosis with necrosis of younger leaves, collectively indicating Se phytotoxicity [29]. Sulphate supply alone produced modest effects; the exception was 0.2 mM sulphate without Se, which reduced leaf weight by 27% relative to the 1 mM control, consistent with the moderate S limitation. These observations place the selenite phytotoxicity threshold between 20 and 80 µM and identify 20 µM as the supply level most favourable to growth.

### 2.5. Free and Protein-Bound Amino Acid Profiles Across Treatments

**Figure 4:**
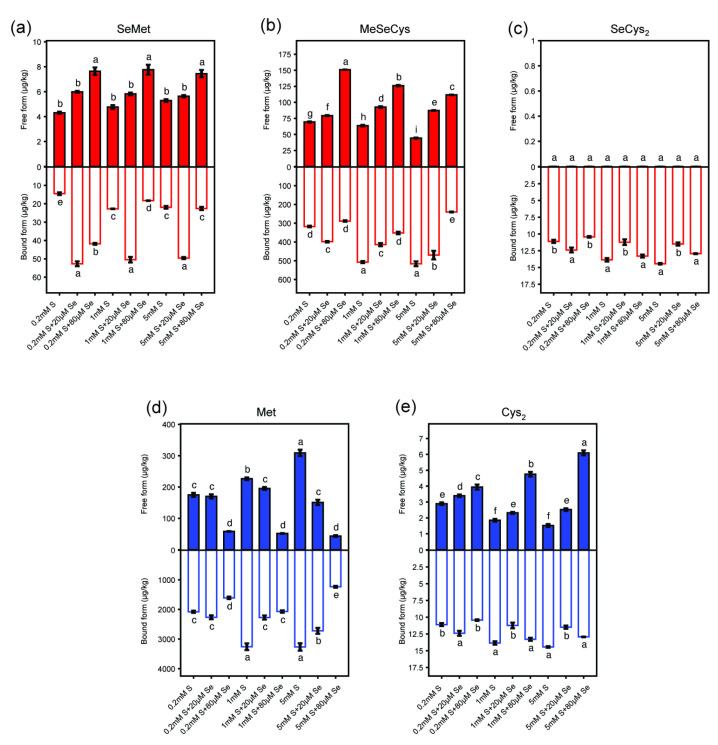
Free and protein-bound SeAA and SAA concentrations in soybean leaves across nine treatments. (**a**) SeMet. (**b**) MeSeCys. (**c**) SeCys_2_. (**d**) Met. (**e**) Cys_2_. Upper bars: free; lower bars: bound. Red bars: Se-containing amino acids; blue bars: S-containing amino acids. Mean ± SD. Different lowercase letters indicate significant differences within each fraction at *p* < 0.05 (Tukey’s HSD).

Free and protein-bound concentrations of SeMet, MeSeCys, SeCys_2_, Met, and Cys_2_ are shown in Figure 4. Free SeCys_2_ remained below the detection limit in every group, whereas bound SeCys_2_ was consistently detected at 10–15 µg kg^−1^ dry weight with limited variation.

Among the quantifiable free SeAAs, concentration rose with selenite supply at every S level, and MeSeCys was the dominant contributor. At 80 µM Se, free MeSeCys reached 150.9, 126.0, and 111.6 µg kg^−1^ at 0.2, 1, and 5 mM S; free SeMet increased from 4.3–5.3 µg kg^−1^ in Se-free controls to 7.2–8.3 µg kg^−1^ at 80 µM Se. Free Met showed the opposite trend, declining from 174–309 µg kg^−1^ in controls to 42–60 µg kg^−1^ at 80 µM Se (75–80% reduction). Free Cys_2_ varied less markedly (1.5–6.3 µg kg^−1^). Two-way ANOVA (Table S6) attributed 87% of variance in the free SeAA pool to Se supply (η^2^ = 0.871) and 83% in the free SAA pool (η^2^ = 0.832), whereas S supply accounted for <6% of variance in each case. The S × Se interaction term captured 7–14%.

The protein-bound fraction displayed a partially different pattern. Bound SeMet peaked at 20 µM Se (49–54 µg kg^−1^) and was lower at 80 µM Se (18–43 µg kg^−1^). Bound MeSeCys showed a similar non-monotonic response, reaching maxima of 400–500 µg kg^−1^ at 20 µM Se. Bound Met and Cys_2_ responded strongly to S supply under Se-free conditions (2080–3360 µg kg^−1^ for Met; 30–45 µg kg^−1^ for Cys_2_) and were substantially reduced by 80 µM Se at every S level. Two-way ANOVA for the bound fractions showed a redistribution of variance relative to the free fractions, with S supply contributing 13–15% (η^2^ = 0.132–0.154) and the S × Se interaction 25–29% (η^2^ = 0.245–0.289).

### 2.6. Stoichiometric Relationships Between SeAA and SAA Pools

**Figure 5:**
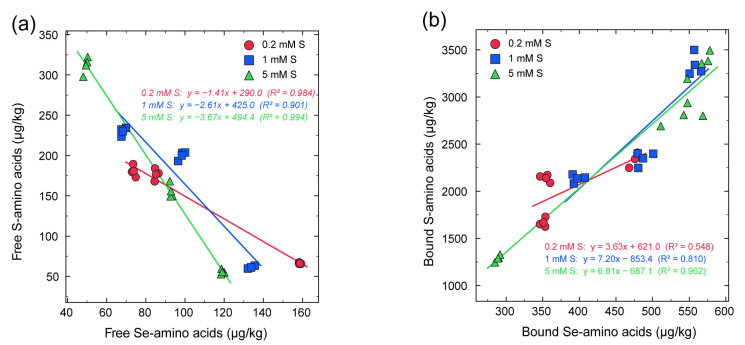
Relationships between SeAA and SAA pools across nine treatment-group means. (**a**) Free fraction. (**b**) Bound fraction. Solid lines: Fitted linear regression; R^2^ and regression equations shown.

Figure 5 summarises the quantitative relationships between total SeAA and SAA pools at the treatment-mean level. In the free fraction (Figure 5a), the two pools were strongly and negatively correlated (R^2^ = 0.83, *p* < 0.001; free SeAAs = −0.36 × free SAAs + 155). Each 1 µg kg^−1^ decrease in free SAAs corresponded, on average, to a 0.36 µg kg^−1^ increase in free SeAAs across the 0.2–5 mM S and 0–80 µM Se range. In the protein-bound fraction (Figure 5b), the relationship reversed: bound SeAAs and SAAs were positively correlated (R^2^ = 0.89, *p* < 0.001; bound SeAAs = 0.14 × bound SAAs + 116), indicating that protein-bound SeAAs accumulated in approximate proportion to the bulk SAA pool rather than substituting for it.

## 3. Discussion

### 3.1. Analytical Value of the Unified Workflow

Three attributes of the workflow underpin the biological resolution achieved. First, MRM detection resolves SeAAs from their S analogues regardless of chromatographic co-elution, as illustrated by the Cys_2_-SeCys_2_ pair (Figure 1), and supports derivatisation-free analysis. This eliminates the procedural variability that accompanies fluorescence-label methods [21,30] and is particularly advantageous for labile Se species whose derivatisation yields are sensitive to buffer composition and reaction time. The five target analytes elute within 1.5 min, and the effective analytical window for the principal organic Se forms closes within this interval, supporting high-throughput quantification in large sample sets typical of biofortification studies. The sensitivity on a five-analyte panel is comparable to that of single-analyte MRM assays targeting SeMet or Met [22,23], but enables direct Se-to-S comparisons within each injection. Within the panel, the LOD of SeMet (0.192 µg/L) is approximately five-fold higher than that of Met (0.036 µg/L). This reflects the intrinsic signal partitioning of polyisotopic Se across its six stable isotopes (only ~49.6% as ^80^Se) [31], together with the well-documented susceptibility of SeMet to oxidation during sample handling and ESI ionisation [32] and the higher chemical background in the SeMet precursor *m*/*z* region. The SeMet LOD nevertheless remains well below the endogenous concentrations measured in soybean leaf samples, and thus does not compromise quantitative reliability.

Second, the UAE step produces free-fraction extracts in which SeAA and SAA recoveries are not differentially biased. Although UAE itself is well established [33,34], its targeted optimisation for simultaneous recovery of free Se and S amino acids from a plant matrix is a useful refinement, ensuring that downstream Se-to-S comparisons reflect in planta pool sizes rather than differential extraction efficiency. The near-coincidence of the two response surfaces on a single optimal condition (Figure 2) and the sub-2% prediction–verification agreement support the robustness of the extraction step. External spike recovery in soybean and *C. violifolia* leaves further returned values of 87.4% to 98.3% across all six analytes (Table S2), confirming that the optimised condition translates to a compositionally distinct leaf matrix. Enzymatic hydrolysis of the same UAE pellet pairs free and bound fractions within each replicate, eliminating sample-level heterogeneity as a confound.

Regarding the bound-fraction workflow, the potential for species transformation during the 4 h enzymatic hydrolysis was mitigated by employing a light-protected Tris-HCl buffer system (pH 8.0) [35,36]. The effectiveness of this approach is supported by the high and symmetric recoveries (86.0–97.6%) observed for all paired Se and S analogues (Table S3). The lack of differential loss or significant interconversion indicates that the enzymatic process preserved the original speciation. Thus, while inherent to such protocols, minor transformations did not compromise the quantitative trends observed across the Se–S treatments.

Third, the workflow offers improved analytical accessibility. HPLC–ICP-MS remains the reference platform [18,19] but its capital cost and specialised requirements concentrate use within dedicated laboratories. Triple-quadrupole MS instrumentation is more broadly distributed, making the present workflow transferable to routine organic Se speciation where element-specific detection is unavailable. Together with the verified stability of speciation through the hydrolysis step, these three attributes collectively provide the analytical foundation on which the biological observations rest, and the workflow is readily extensible to Se–S studies in other leafy crops.

### 3.2. Direct Quantitative Evidence for Se–S Metabolic Interaction

Competitive uptake and co-assimilation of Se and S in plants have been inferred primarily from transport-physiology evidence and single-analyte measurements. Sulphate transporters mediate selenate uptake [16,37,38]; S deficiency up-regulates SULTR expression and thereby enhances Se uptake [13,14]; and Se and S share the downstream assimilation enzymes generating Cys, Met, and their Se analogues [10,11,12,39]. Because the present experiment supplied Se as selenite rather than selenate, the observed competition is unlikely to originate at the transporter level; selenite enters root cells through phosphate transporters and aquaporins rather than SULTRs [40,41]. The negative free-pool correlation is most readily interpreted as arising from enzymatic competition downstream of uptake, where selenite-derived selenide and sulphate-derived sulphide converge on ATP sulfurylase, cysteine synthase, and methionine synthase [10,11,12], although contributions from compartmentation, differential turnover, or redox-driven glutathione demand [42,43] cannot be excluded on the basis of the present steady-state data. The present workflow provides quantitative evidence consistent with this downstream interaction by reporting free and bound concentrations of three SeAAs and two SAAs simultaneously across nine defined Se–S supply combinations, resolving individual analyte response patterns that are obscured when only total Se or S is reported.

The reciprocal response of free MeSeCys and free Met across the Se gradient is a prominent molecular-level pattern consistent with competitive Se-S assimilation. As selenite rose from 0 to 80 µM, free MeSeCys increased approximately tenfold at every sulphate level while free Met fell by 75–80%. A smaller but directionally consistent shift occurred between free SeMet and Met. The near-linear negative correlation (R^2^ = 0.83, Figure 5a) suggests that the two pools may exchange along a quantitatively predictable trajectory rather than through an all-or-nothing switch, consistent with redirected Cys and Met biosynthetic flux into the Se analogues with concomitant depletion of the free SAA pool [10,11,12].

The reversal in the protein-bound fraction (R^2^ = 0.89, Figure 5b) adds a complementary layer of evidence. Bound SeAAs and SAAs accumulate in approximate proportion, consistent with the well-documented non-discriminatory incorporation of SeMet at Met residues by Met-tRNA synthetase [44]. Under this mechanism, any treatment supporting robust protein synthesis would also support incorporation of both species classes in roughly fixed stoichiometry, so that bound Se represented by SeMet tracks Met abundance in the total amino acid pool [26]. An alternative and not mutually exclusive contribution is active de novo SeMet synthesis through the S assimilation pathway followed by translational incorporation. The two routes differ in their rate-limiting step: non-specific incorporation is limited by the SeMet-to-Met ratio in the free pool weighted by translation rate, whereas de novo synthesis is limited by biosynthetic flux through the assimilation pathway. The present steady-state dataset cannot distinguish which route dominates, and resolving their relative contributions would require isotopic pulse-chase labelling with stable Se tracers or targeted perturbation of branch-point enzymes such as selenocysteine methyltransferase. Until such experiments are conducted, the free and bound designations reported here should be understood as operationally defined by the sequential extraction procedure rather than as thermodynamically discrete compartments. The larger S × Se interaction effect sizes in bound fractions (η^2^ = 0.245–0.289) relative to free fractions (η^2^ = 0.071–0.135) are consistent with a protein-level interpretation in which protein incorporation integrates upstream fluctuations in both S and Se metabolism, whereas free-pool composition is dominated by immediate Se supply. The bell-shaped response of bound SeMet and MeSeCys, peaking at 20 µM Se with reduced values at 80 µM, mirrors the biomass response (Figure S3) and is consistent with reduced protein synthesis under phytotoxic conditions [29].

The ANOVA decomposition highlights an important nuance. Se supply dominated variance in both free and bound pools (η^2^ = 0.83–0.87 for free fractions), while S as a main effect contributed <6%. The S × Se interaction term, however, captured 7–14% in free pools and 25–29% in bound pools, suggesting that S modulates the metabolic direction of Se assimilation rather than independently determining amino acid concentrations. This pattern contrasts with studies under S deficiency, where S limitation strongly drives Se accumulation in Arabidopsis [13] and wheat [14]. The difference likely reflects the narrower and generally sufficient S range (0.2–5 mM) employed here, which did not impose a severe limitation except at the lowest level. The Se-S interaction demonstrated is thus best characterised as metabolic-level modulation within the S-sufficient range, rather than transport-level competition that dominates under S depletion.

### 3.3. Implications for Selenium Biofortification of Soybean

The approval of soy protein as a permitted carrier for selenium fortification under the Chinese national standard GB 14880-2012 provides regulatory context for investigating selenium biofortification strategies in soybean [24]. Among the nine treatments, 20 µM selenite consistently defined the upper boundary of a beneficial supply range as assessed by leaf-level endpoints. At this supply level, plants developed the most extensive root systems, maintained leaf biomass comparable to Se-free controls, and produced the highest protein-bound SeMet and MeSeCys (49–54 and 400–500 µg kg^−1^ dry weight, respectively). Within this Se level, 5 mM S with 20 µM Se (Group F) yielded the highest total bound SeAA content (542 µg kg^−1^) while preserving leaf weight at 1.27 g per plant, indistinguishable from the Se-free control. The 1 mM S with 20 µM Se (Group E) provided a comparable outcome at slightly higher biomass. Together these conditions define a preliminary favourable window at the leaf level spanning 1–5 mM S at 20 µM Se, although its applicability to seed Se accumulation remains to be established.

The rationale underlying this window is consistent with the Se-S interactions characterised above. At 20 µM Se, selenite supply drives measurable protein-level incorporation of the two bioavailable organic Se forms preferentially accumulated in soybean storage proteins [26] without reaching the concentration at which competitive displacement of the free SAA pool is associated with phytotoxicity and reduced protein synthesis [29]. The enhanced root development and modest biomass gain observed at 20 µM Se are consistent with reports that sub-toxic Se can promote antioxidant defence and photosynthetic performance in various crop species [9,16]. The precise mechanism in soybean root tissue, however, is not investigated here and warrants further study. The intact free-Met pool in Groups E/F (109–174 µg kg^−1^) suggests that S-assimilation capacity was not overwhelmed, thereby sustaining substrate availability for the proportional bound-SeMet incorporation described by the positive R^2^ = 0.89 relationship (Figure 5b).

By contrast, the condition maximising free organo-Se (Group G: 0.2 mM S; 80 µM Se) achieved elevated free MeSeCys only at the cost of severe phytotoxicity and paradoxically lower bound SeAA content, rendering it unsuitable as a production protocol. These leaf-level observations provide a starting point for the design of seed-focused experiments aimed at producing Se-enriched soy-protein ingredients [6,7]. It must be emphasised, however, that leaf SeAA distribution may differ substantially from that in seed protein. MeSeCys was the dominant free SeAA in the present leaf data, consistent with active Se methylation in foliar tissue [10,11], whereas SeMet constitutes >80% of the Se in the glycinin fraction of soybean seed [26]. Because Se speciation is remodelled during phloem loading, long-distance translocation, and seed filling [6,10], the operating window identified here is defined by leaf-level endpoints, and its translation to seed protein-bound SeAA content cannot be assumed without direct measurement. The conditions maximising leaf-bound MeSeCys may not coincide with those maximising seed SeMet, as the two endpoints are separated by translocation, remobilisation, and differential incorporation during seed filling. Any biofortification recommendations derived from these data should therefore be regarded as provisional hypotheses requiring seed-level validation. Direct confirmation in a seed-focused experiment is an essential next step before agronomic recommendations can be formulated.

## 4. Materials and Methods

### 4.1. Chemicals and Reagents

L-Methionine (99%) and L-selenomethionine (98%) were obtained from J&K Scientific (Beijing, China). L-Cystine (99.5%), L-selenocystine (98%), and selenoethionine (98%) were from Macklin Biochemical (Shanghai, China), and Se-methyl-L-selenocysteine (≥98%) from Aladdin Biochemical (Shanghai, China). HPLC-grade methanol (Merck, Darmstadt, Germany) and formic acid (Thermo Fisher Scientific, Waltham, MA, USA) were used for mobile phases. Sodium selenite (99%, Yuanye Bio-Technology, Shanghai, China), anhydrous sodium sulphate, nitric acid, and sulphuric acid (analytical grade, Sinopharm Chemical Reagent, Shanghai, China) were used for hydroponic culture and elemental analysis. The sulphur-free Hoagland nutrient-solution base was from Coolaber (Beijing, China). In terms of trypsin (1:4000 activity ratio, Yuanye Bio-Technology), proteinase K (43.6 U mg^−1^), and pronase E (7320 U g^−1^), the latter two from Solarbio (Beijing, China) were used for enzymatic hydrolysis in 50 mM Tris-HCl buffer at pH 8.0. Ultrapure water (18.2 MΩ·cm) was produced on a Milli-Q Synthesis system (Millipore, Bedford, MA, USA). Individual stock solutions were prepared in ultrapure water at 1.0 g L^−1^, stored at −20 °C in amber vials, and used within three months. Working calibration solutions were freshly prepared by serial dilution with 0.1% (*v*/*v*) aqueous formic acid on the day of analysis.

### 4.2. HPLC–MS/MS Analysis

Separation and detection were performed on a SCIEX QTRAP 6500+ tandem mass spectrometer coupled to an ExionLC AD UHPLC (SCIEX, Framingham, MA, USA). Analytes were resolved on a Waters ACQUITY UPLC BEH C18 column (130 Å, 2.1 mm × 50 mm, 1.7 µm) maintained at 40 °C. Mobile phase A was 0.1% (*v*/*v*) aqueous formic acid; mobile phase B was 0.1% formic acid in methanol. The flow rate was 0.200 mL min^−1^ with a 2 µL injection volume. The gradient (A/B, *v*/*v*) was 95/5 from 0 to 2 min, ramped linearly to 65/35 at 4 min, returned to 95/5 at 5 min, and held until 7 min for re-equilibration.

The mass spectrometer operated in positive-ion electrospray ionisation mode with MRM acquisition. Source parameters: Curtain gas: 35 psi, ion-spray voltage: 5500 V, source temperature: 450 °C, nebuliser gas (GS1) and heater gas (GS2): 45 psi each, and collision gas: medium. Two MRM transitions per analyte (one quantifier, one qualifier) were monitored with compound-specific declustering potentials and collision energies optimised by direct infusion (Table S1). Data were acquired and integrated with Analyst 1.7 and MultiQuant 3.0.3 (SCIEX).

### 4.3. Method Validation

Linearity, sensitivity, precision, and trueness were assessed following Eurachem guidelines [27]. Calibration curves were constructed from seven mixed-standard levels and fitted by weighted least-squares regression (1/x weighting). LOD and LOQ were defined as concentrations yielding S/N ratios of 3 and 10 in spiked matrix extracts. Intra-day and inter-day precision were assessed at three concentration levels (1, 10, and 100 µg L^−1^ for SeAAs and Cys_2_; 50, 500, and 5000 µg L^−1^ for Met), with six replicate injections within one day and on three consecutive days. Trueness was determined by spike recovery in the pooled leaf matrix at three levels per analyte (*n* = 6 per level). Matrix effects were estimated from slope ratios of matrix-matched to solvent-only calibration curves [28]. System stability during analysis of study samples was monitored by injecting pooled QC samples to bracket each analytical sequence, following Eurachem guidance on internal quality control [27]. The pooled QC sample was prepared by combining equal aliquots of all leaf extracts from the factorial experiment. Study-sample injection order was randomised within each sequence to distribute any temporal drift across treatment groups. Solvent blanks were injected after each QC injection to monitor carryover. Peak area and retention time relative standard deviations across the QC injections, together with carryover assessment, are summarised in Table S7.

### 4.4. Hydroponic Experiment and Leaf Sampling

Soybean (*Glycine max* L. Merr.) cultivar Dongnong 690 (Northeast Agricultural University, Harbin, China) was used throughout. Seeds were surface-sterilised in 1% (*v*/*v*) NaClO for 10 min, rinsed, soaked for 24 h, and germinated on moist cotton at 25 °C in the dark for 3 d. Uniform V1-stage seedlings were transferred to nine 6 L opaque hydroponic containers (four plants per container) filled with modified sulphur-free Hoagland solution amended with treatment-specific Se and S concentrations. The growth chamber was maintained at 16 h light/25 °C and 8 h dark/20 °C, with 65 ± 5% relative humidity and ~400 µmol m^−2^s^−1^ PPFD at canopy height from white LED panels. Solution pH was adjusted daily to 6.0 ± 0.2, nutrient solution was renewed every 3 d, and continuous aeration was supplied by aquarium pumps.

A 3 × 3 factorial design combined three sulphate levels (0.2, 1.0, and 5.0 mM SO_4_^2−^ as Na_2_SO_4_) with three selenite levels (0, 20, and 80 µM Se(IV) as Na_2_SeO_3_), giving nine treatment groups labelled a–i (Table S5). Each treatment comprised twelve biological replicates, with three plants pooled per replicate. After 28 d, fully expanded leaves were harvested, rinsed three times with ultrapure water, blotted dry, flash-frozen in liquid nitrogen, freeze-dried to constant mass, and ground to fine powder in a ball mill (MM 400, Retsch, Haan, Germany). Powders were stored at −80 °C until analysis. Leaves were chosen as the analytical matrix because they are an important site of Se assimilation, where selenite is reduced and converted to organic SeAAs through the S assimilation pathway [10,11].

### 4.5. Box–Behnken Optimisation of Ultrasound-Assisted Extraction

Free amino acids were recovered from freeze-dried leaf powder by UAE. Three extraction parameters were jointly optimised by response-surface methodology using a Box–Behnken design with three levels per factor [45,46]: ultrasonication time (A, 40–60 min), temperature (B, 40–60 °C), and solvent-to-sample ratio (C, 8–12 mL g^−1^). The design comprised 17 runs including five centre-point replicates in randomised order. For each run, 0.1 g powder was suspended in the assigned volume of 0.1% (*v*/*v*) aqueous formic acid, sonicated in a KQ-500DE ultrasonic bath (Kunshan Ultrasonic Instruments, 500 W, 40 kHz), centrifuged at 10,000× *g* for 10 min at 4 °C, passed through a 0.22 µm PTFE syringe filter, and analysed by HPLC–MS/MS.

Two responses were measured simultaneously: Y_1_ (total free organo-Se = SeMet + SeCys_2_ + MeSeCys, µg kg^−1^ dry weight) and Y_2_ (total free SAAs = Met + Cys_2_, µg kg^−1^ dry weight). Monitoring both responses was essential because divergent recovery efficiencies would have conflated biological variation with an analytical artefact. A single jointly optimal condition was identified using the Design-Expert desirability function. Each response was fitted to a second-order polynomial by least-squares regression [45,46]. Model adequacy was assessed by ANOVA (significance at *p* < 0.05 with non-significant lack of fit at *p* > 0.05), and the predicted optimum was confirmed by triplicate verification. All design generation, regression, and response-surface plotting steps were performed in Design-Expert 13 (Stat-Ease, Minneapolis, MN, USA).

### 4.6. Enzymatic Hydrolysis of the Protein-Bound Fraction

After UAE centrifugation, the pellet was washed three times with ultrapure water; resuspended in 30 mL of 50 mM Tris-HCl buffer (pH 8.0); sonicated for 5 min to disperse; and digested with 10 mg each of trypsin, proteinase K, and pronase E (combined enzyme-to-substrate ratio: ~15%) at 37 °C, with 100–120 r min^−1^ for 4 h. The hydrolysate was centrifuged at 10,000× *g* for 10 min at 4 °C, filtered through a 0.22 µm PTFE membrane, and analysed on the same HPLC–MS/MS platform. Using the UAE-derived pellet ensured that free and bound fractions were defined on a single sample aliquot, enabling direct stoichiometric comparison within each replicate.

The three-protease cocktail in Tris-HCl was adopted from established Se-speciation protocols rather than being optimised de novo. This combination releases Se from plant biomass at recoveries above 85%, well above the 10–20% achievable by solvent leaching alone, across diverse plant matrices [47,48]. Tris-HCl preserves all major Se species during incubation, offering a stability advantage over citrate or phosphate buffers [35], and the 4 h incubation at 37 °C falls within the plateau region of SeAA release that was previously identified [36]. To externally verify the integrity of speciation across the enzymatic hydrolysis step, spike-recovery experiments were performed in soybean (*G. max*) leaves and leaves of the Se-hyperaccumulator *C. violifolia*. Six analytes were each spiked into pre-hydrolysis residues (*n* = 3 replicates per analyte per matrix), processed through the standard three-protease digestion procedure, and quantified against matrix-matched calibration.

### 4.7. Statistical Analysis

All data are expressed as mean ± standard deviation. Normality and variance homogeneity were assessed by the Shapiro–Wilk and Levene tests before parametric analysis. Treatment differences were evaluated by two-way ANOVA with Se, S, and their interaction as fixed factors, followed by Tukey’s HSD post hoc comparisons at *p* < 0.05. Relationships between SeAA and SAA pools within free and bound fractions were characterised by Pearson correlation and simple linear regression across the nine treatment groups, with R^2^ reported as an index of explanatory power. All analyses were performed in IBM SPSS Statistics 27.0 (IBM, Armonk, NY, USA).

## 5. Conclusions

A unified, derivatisation-free HPLC–MS/MS workflow has been developed and validated for simultaneous quantification of five SeAAs and SAAs in soybean leaves. The five target analytes elute within a 1.5 min window, enabling high-throughput organic Se quantification. The method provides sub-µg L^−1^ detection limits, intra-day RSDs below 5%, and spike recoveries of 92–117%. Box–Behnken optimisation of UAE identified a single condition (10 mL g^−1^, 50 min, 50 °C) at which free organo-Se and free SAA pools were jointly maximised, and enzymatic hydrolysis of the UAE residue extended the platform to the protein-bound fraction. Because triple-quadrupole MS is more widely available than HPLC-ICP-MS, the workflow is suited to routine organic Se speciation outside specialist laboratories. Although quantification relies on matrix-matched external calibration rather than stable-isotope-labelled internal standards, incorporation of isotope dilution for Met and SeMet, for which commercial standards are available, would further strengthen traceability. Application to soybean under a 3 × 3 Se–S factorial regime provided direct evidence of Se-S metabolic interaction: free SeAA and SAA pools were negatively correlated (R^2^ = 0.83), consistent with competitive assimilation, and bound pools positively correlated (R^2^ = 0.89), consistent with proportional protein-level incorporation. A regime of 1–5 mM sulphate with 20 µM selenite emerged as a preliminarily favourable leaf-level operating window that combined elevated bound organo-Se with near-normal growth. As the current data reflect steady-state pool sizes rather than dynamic metabolic fluxes, and leaf SeAA profiles may not directly predict seed Se speciation, further verification through flux-resolved and seed-focused experiments is necessary before these findings can be translated into biofortification practice.

## Figures and Tables

**Table 1:** Analytical performance of the HPLC–MS/MS method in soybean leaf matrix.

Analyte	LDR (μg/L)	R^2^	LOD (μg/L)	LOQ (μg/L)	Intra-Day Precision (RSD, %)	Inter-Day Precision (RSD, %)	Spike Recovery (%)	Matrix Effect (%)
Met	50–5000	0.9999	0.036	0.121	0.87/0.82/1.66	2.79/2.38/2.03	113.2/108.8/95.1	92.4
MeSeCys	1–400	0.9997	0.161	0.538	2.84/2.88/1.36	7.14/2.87/2.26	94.0/102.5/102.4	88.6
SeMet	1–400	0.9998	0.192	0.641	3.34/3.27/2.39	6.36/3.59/2.16	114.4/115.2/115.4	91.2
SeEt	1–400	0.9995	0.556	1.852	4.16/4.24/4.05	6.24/5.78/4.00	98.3/94.6/105.1	86.5
Cys2	1–400	0.9998	0.064	0.212	2.61/1.05/1.09	4.46/2.51/1.67	114.8/117.4/92.3	82.3
SeCys2	1–400	0.9999	0.125	0.417	2.76/3.59/2.00	8.06/3.09/1.69	113.0/104.7/94.6	89.1

LDR, linear dynamic range. LOD, limit of detection (S/N = 3). LOQ, limit of quantification (S/N = 10). Precision reported as RSD (%) at three levels (*n* = 6). Spike recovery (%): Mean of six replicates at three levels. Matrix effect (%): Slope ratio of matrix-matched to solvent-only curves; <100% = ion suppression, >100% = ion enhancement.

## Data Availability

The data presented in this study are available on request from the corresponding author.

## Supplementary Materials

The following supporting information can be downloaded at https://www.mdpi.com/article/10.3390/molecules31111780/s1: Table S1. Optimised MRM transitions and compound-dependent parameters for the six analytes on the SCIEX QTRAP 6500+; Figure S1. Product ion MS/MS spectra of the target selenium-containing amino acids and their sulphur analogues; Table S2. Recovery of six selenium- and sulphur-containing amino acids spiked in *G. max* and *C. violifolia* leaves before UAE; Table S3. Recovery of six protein-bound amino acids in *G. max* and *C. violifolia* leaves via enzymatic hydrolysis; Table S4. Box–Behnken design matrix and responses; Table S5. ANOVA summary for the Box–Behnken polynomial models; Figure S2. Two-dimensional contour plots of the Box–Behnken design for UAE optimisation; Figure S3. Leaf fresh weight per plant; Table S6. Two-way ANOVA results for Se, S, and S × Se effects on free and bound amino acid content; Figure S4. Free and protein-bound SeAA and SAA concentrations in soybean leaves across nine treatments; Table S7. Instrumental stability assessment via peak area and retention time of repeated injections.
