# Supplementary material for: Development and Optimisation of an HPLC–MS/MS Workflow for Profiling Selenium and Sulphur Amino Acids in Soybean Leaves and Investigation of Se–S Metabolic Interactions
Source: Molecules. 2026 May 22;31(11):1780. doi: 10.3390/molecules31111780 (PMC13258760; doi:10.3390/molecules31111780)
Supplement: Supplementary file 1 [file molecules-31-01780-s001.zip › molecules-4303481-supplementary.pdf]

Development and Optimization of an HPLC–MS/MS Workflow for Profiling Selenium and Sulfur Amino Acids in Soybean Leaves and Investigation of Se–S Metabolic Interactions

Xiaohui Cai [1], Jun Men [2], Qingwu Yang [1] , Yili Hu [1,*] and Zhixian Qiao [2,*]

1.School of Modern Industry for Selenium Science and Engineering, Wuhan Polytechnic University, Wuhan 430048, China

2.Analysis and Testing Center, Institute of Hydrobiology, Chinese Academy of Sciences, Wuhan 430072, China

*: Corresponding author Email: huyili@whpu.edu.cn；qzhxian@ihb.ac.cn

Table S1. Optimised MRM transitions and compound-dependent parameters for the six analytes on the SCIEX QTRAP 6500+. Q: quantifier transition. DP: declustering potential. CE: collision energy.

Analyte	Precursor (m/z)	Product (m/z)	DP (V) Q	DP (V) qualifier	CE (V) Q	CE (V) qualifier
Met	150	104.0 (Q) / 133.0	11	17	17	27
MeSeCys	184	139.0 (Q) / 167.0	26	15	19	10
SeMet	198	109.0 (Q) / 181.0	15	25	20	10
SeEt	212	123.0 (Q) / 195.0	18	10	20	10
Cys_2	241	120.0 (Q) / 152.0	10	10	20	15
$SeCys_2$	335	246.0 (Q) / 88.0	20	20	15	25

Figure S1. Product ion MS/MS spectra of the target selenium-containing amino acids and their sulfur analogues. The characteristic fragments used for MRM transitions are labeled, confirming the structural identity of (a) L-Cystine, (b) L-Methionine, (c) L-selenocystine, (d) Selenoethionine, (e) Se-methyl-L-selenocysteine, and (f) L-selenomethionine. The spectra were obtained at optimized collision energies as specified in Table S1.

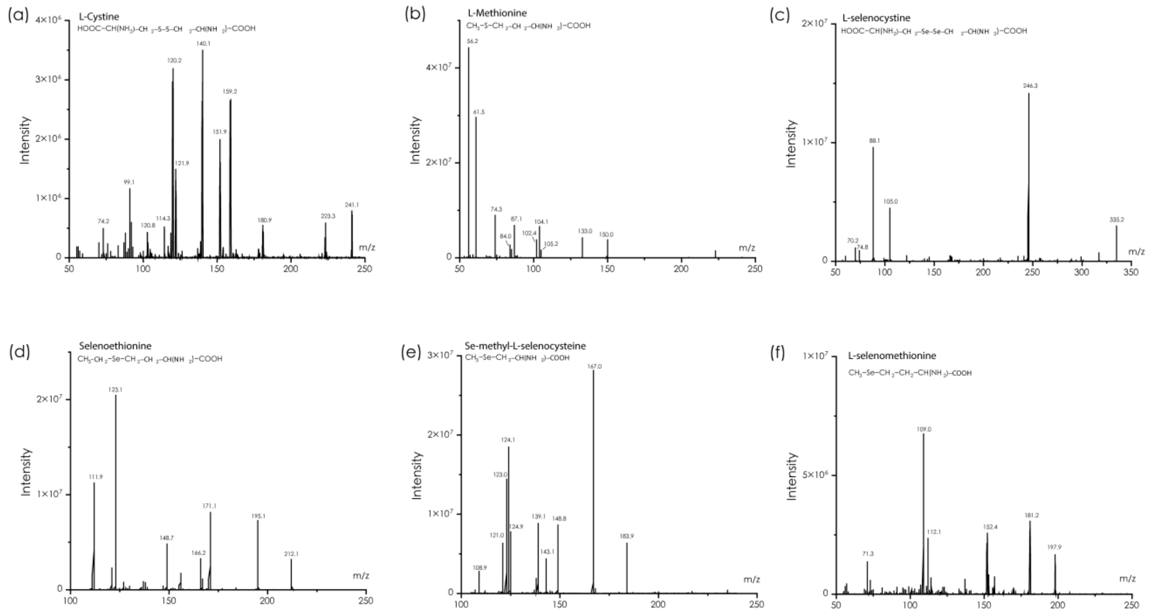

Table S2. Recovery of six selenium- and sulfur-containing amino acids spiked in *G. max* and *C. violifolia* leaves before UAE.

Analyte	Matrix	Endogenous (μg/kg)	Spiked (μg/kg)	Total Measured (μg/kg)	Recovery (%)	RSD (n=3, %)
Met	*G. max*	155.2	100	251.5	96.3	3.2
	C. violifolia	182.45	100	280.12	97.67	2.8
SeMet	*G. max*	5.32	10	14.85	95.3	4.5
	C. violifolia	124.6	100	218.9	94.3	3.9
MeSeCys	*G. max*	12.15	20	31.25	95.5	4.1
	C. violifolia	455.8	200	652.4	98.3	3.5
Cys2	*G. max*	4.28	10	13.02	87.4	5.2
	C. violifolia	6.15	10	15.18	90.3	4.8
SeCys2	*G. max*	12.4	10	21.25	88.5	5.8
	C. violifolia	15.12	10	24.08	89.6	6.1
SeEt	*G. max*	< LOD	10	9.42	94.2	4.9
	C. violifolia	< LOD	10	9.65	96.5	5.3

Table S3. Recovery of six protein-bound amino acids in *G. max* and *C. violifolia* leaves via enzymatic

hydrolysis.

Analyte	Matrix	Endogenous (μg/kg)	Spiked (μg/kg)	Total Measured (μg/kg)	Recovery (%)	RSD (n=3, %)
Met	*G. max*	2150.4	1000	3118.5	96.81	3.5
	C. violifolia	2480.15	1000	3455.6	97.55	3.2
SeMet	*G. max*	52.15	50	99.85	95.4	4.8
	C. violifolia	1150.4	500	1638.2	97.56	4.1
MeSeCys	*G. max*	425.6	200	618.4	96.4	4.5
	C. violifolia	5800.2	2000	7720.5	96.02	4.9
Cys2	*G. max*	35.12	20	52.32	86	5.5
	C. violifolia	42.85	20	60.27	87.1	5.1
SeCys2	*G. max*	12.8	10	22.15	93.5	6.2
	C. violifolia	14.55	10	23.95	94	5.8
SeEt	*G. max*	< LOD	10	9.55	95.5	5.9
	C. violifolia	< LOD	10	9.62	96.2	6.1

Table S4. Box–Behnken design matrix and responses. A: sonication time. B: sonication temperature. C: solvent-to-sample ratio. Y_1: total free organo-Se. Y_2: total free SAAs.

Std	Run	*A* (min)	*B* (°C)	*C* (mL g^{-1})	Y_1 (ug kg^{-1})	Y_2 (ug kg^{-1})
1	5	40	40	10	21.778	41.2
2	8	60	40	10	22.298	40.57
3	2	40	60	10	22.355	40.09
4	6	60	60	10	22.079	41.65
5	13	40	50	8	20.449	38.61
6	11	60	50	8	19.317	38.85
7	4	40	50	12	22.183	41.92
8	14	60	50	12	22.338	41.41
9	17	50	40	8	19.934	36.53
10	7	50	60	8	19.02	37.13
11	15	50	40	12	22.123	41.65
12	12	50	60	12	22.362	41
13	9	50	50	10	24.596	45.09
14	16	50	50	10	25.016	44.01

15	3	50	50	10	24.859	43.99
16	1	50	50	10	25.203	44.01
17	10	50	50	10	25.542	43.99

Table S5. ANOVA summary for the Box–Behnken polynomial models. Regression coefficients, F-values, and p-values for each term; model-fit statistics. Significant terms ($p < 0.05$) in bold.

**Source**	**Y_1 (sulfur amino acids)**			**Y_2 (organo-Se)**		
	SS	***F***	***p***	***SS***	***F***	***p***
Model	97.29	32.23	< 0.0001 ***	65.96	56.75	< 0.0001 ***
A (time)	0.054	0.16	0.699	0.067	0.52	0.4942
B (temp.)	8.0×10^{-4}	2.4×10^{-3}	0.9624	0.013	0.097	0.7642
C (ratio)	27.6	82.3	< 0.0001 ***	13.23	102.41	< 0.0001 ***
AB	1.2	3.58	0.1006	0.16	1.23	0.3047
AC	0.14	0.42	0.5379	0.41	3.21	0.1165
BC	0.39	1.16	0.3163	0.33	2.57	0.1527
A^2	5.19	15.47	0.0056 **	7.69	59.58	0.0001 ***
B^2	20.94	62.44	< 0.0001 ***	10.3	79.74	< 0.0001 ***
C^2	35.66	106.33	< 0.0001 ***	28.89	223.73	< 0.0001 ***
Residual	2.35	—	—	0.9	—	—
Lack of fit	1.4	1.96	0.2622 ns	0.4	1.03	0.4672 ns
Pure error	0.95	—	—	0.51	—	—
R^2		0.9764			0.9865	
Adj R^2		0.9461			0.9691	
Pred R^2		0.7608			0.8936	
Adeq. precision		16.44			21.03	

Figure S2. Two-dimensional contour plots of the Box–Behnken design for UAE optimization. The plots (a–f) illustrate the interactive effects of ultrasonication time, temperature, and solvent-to-sample ratio on the combined desirability response. The optimized regions (darker areas) correspond to the 3D surfaces presented in Figure 2 of the main manuscript.

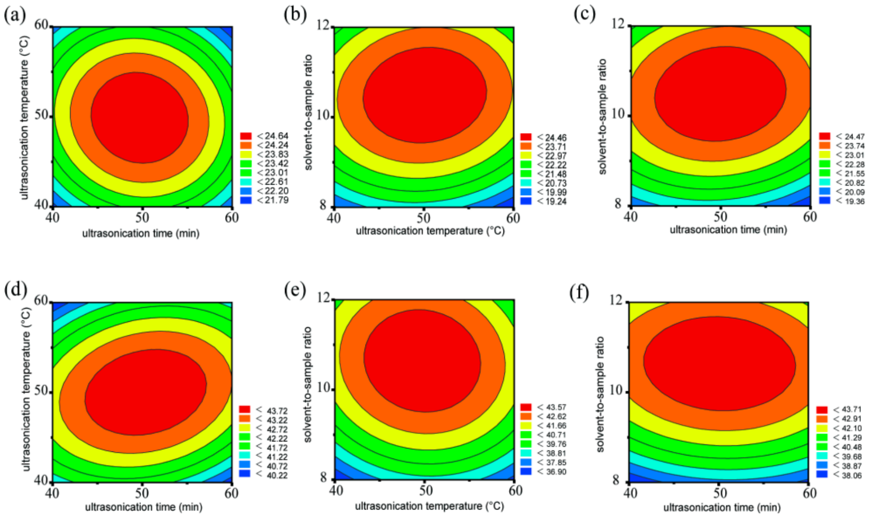

Figure S3. Leaf fresh weight per plant. Mean ± SD; different letters indicate significant differences at $p < 0.05$ (Tukey's HSD).

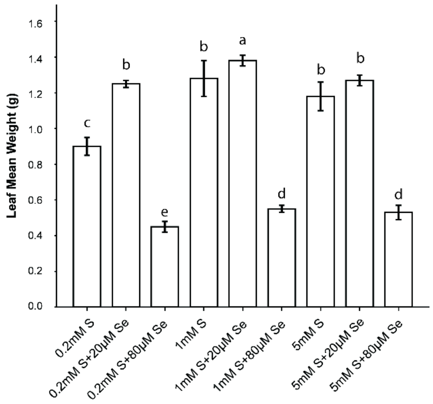

Table S6. Two-way ANOVA results for Se, S, and S × Se effects on free and bound amino acid content. SS: sum of squares. η^2: partial eta-squared. n = 4 per treatment (N = 36). Interaction rows in bold.

Variable	Source	df	SS	F	p	η²
**Free Se-AAs**	S	2	2214.1	1095.7	< 0.001	0.059
	Se	2	32776.1	16219.4	< 0.001	0.871
	**S × Se**	4	2625.6	**649.6**	**< 0.001**	**0.07**
	Residual	27	27.3			
**Free S-AAs**	S	2	6929	95	< 0.001	0.029
	Se	2	199602	2736.6	< 0.001	0.832

	**S × Se**	4	32365.1	**221.9**	**< 0.001**	**0.135**
	Residual	27	984.7			
**Bound Se-AAs**	S	2	51899.4	224.5	< 0.001	0.154
	Se	2	184957.2	800.2	< 0.001	0.548
	**S × Se**	4	97627.7	**211.2**	**< 0.001**	**0.289**
	Residual	27	3120.2			
**Bound S-AAs**	S	2	2064578	171.3	< 0.001	0.132
	Se	2	9598682	796.2	< 0.001	0.613
	**S × Se**	4	3843597	**159.4**	**< 0.001**	**0.245**
	Residual	27	162743			

Figure S4. Total free and protein-bound SeAA and SAA pools across the nine treatments. Upper bars: free fraction; lower bars: protein-bound fraction. Red bars: total Se-containing amino acids (SeMet + MeSeCys + SeCys2); blue bars: total S-containing amino acids (Met + Cys2). Mean ± SD; different lowercase letters indicate significant differences within each fraction at $p < 0.05$ (Tukey's HSD).

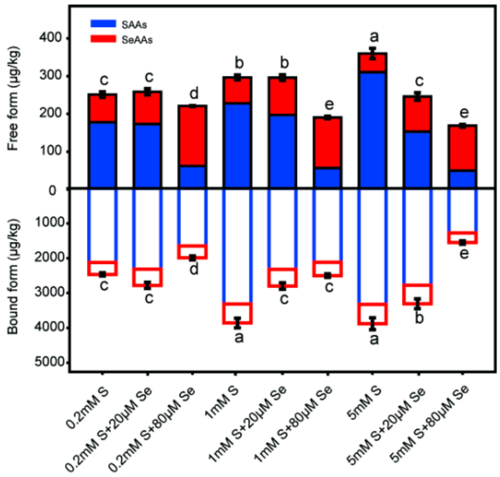

Table S7. Instrumental stability assessment via pooled-QC peak area and retention time across the analytical sequence.

**Analyte**	**Start of sequence**		**Midpoint of sequence**		**End of sequence**		**RSD**	
	**Peak-area**	**RT(min)**	**Peak-area**	**RT(min)**	**Peak-area**	**RT(min)**	**Area （%）**	**RT （%）**
Cys_2	125430	0.659	128950	0.666	123660	0.639	7.6	2.2
$SeCys_2$	45820	0.669	46780	0.671	45110	0.649	6.6	1.8

MeSeCys	89240	0.880	87550	0.848	90880	0.869	6.7	1.9
Met	356400	1.096	348200	1.064	359100	1.094	2.7	1.7
SeMet	18560	1.344	19220	1.303	18330	1.368	5.0	2.5
SeEt	9540	2.801	9210	2.783	9780	2.910	6.9	3.0
